# Evaluation of Vitamin E Isoforms in Placental Tissue and Their Relationship with Maternal Dietary Intake and Plasma Concentrations in Mother–Infant Dyads

**DOI:** 10.3390/antiox12101797

**Published:** 2023-09-24

**Authors:** Ishani Jhamb, Alyssa Freeman, Michelle R. Lotfi, Matthew VanOrmer, Corrine Hanson, Ann Anderson-Berry, Melissa Thoene

**Affiliations:** 1 Department of Pediatrics, Division of Neonatology, University of California San Diego, San Diego, CA 92037, USA; 2 Department of Pediatrics, University of Nebraska Medical Center, Omaha, NE 68198, USA; 3 College of Allied Health Professions, University of Nebraska Medical Center, Omaha, NE 68198, USA

**Keywords:** tocopherol, pregnancy, placenta, vitamin E

## Abstract

α-tocopherol is a vitamin E isoform with potent antioxidant activity, while the γ-tocopherol isoform of vitamin E exerts more pro-inflammatory effects. In maternal–fetal environments, increased plasma α-tocopherol concentrations are associated with positive birth outcomes, while higher γ-tocopherol concentrations are linked with negative pregnancy outcomes. However, little is known about tocopherol concentrations in placental tissue and their role in modulating placental oxidative stress, a process that is implicated in many complications of pregnancy. The objectives of this research are to evaluate the concentrations of α- and γ-tocopherol in placental tissue and assess relationships with maternal and umbilical cord plasma concentrations. A total of 82 mother–infant dyads were enrolled at the time of delivery, and maternal and umbilical cord blood samples and placenta samples were collected. α- and γ-tocopherol concentrations in these samples were analyzed by high-performance liquid chromatography (HPLC). γ-tocopherol concentrations demonstrated significant, positive correlations among all sample types (*p*-values < 0.001). Placental tissue had a significantly lower ratio of α:γ-tocopherol concentrations when compared to maternal plasma and umbilical cord plasma (2.9 vs. 9.9 vs. 13.2, respectively; *p* < 0.001). Additional research should explore possible mechanisms for tocopherol storage and transfer in placental tissue and assess relationships between placental tocopherol concentrations and measures of maternal–fetal oxidative stress and clinical outcomes of pregnancy.

## 1. Introduction

Altered nutrition or metabolism during fetal development lays the foundation for newborn infants and predisposes them to conditions throughout adulthood [1]. Maternal nutrition evidently has an impact on fetal nutrition and growth, and our group has previously identified relationships between fat-soluble nutrient concentrations in the mother and infant [2,3,4]. Vitamin E is a unique fat-soluble nutrient that exists in eight isoforms, with α- and γ-tocopherol being the most well-studied isoforms due to their high concentrations in the human diet and biological tissues. Additionally, α-tocopherol meets the full criteria for classification as an antioxidant, a compound found in the typical human diet that prevents cellular damage caused by reactive oxygen species [5]. Because of this ability to reduce oxidative stress, α-tocopherol has been examined in many aspects of antenatal and postnatal development. For example, lower maternal α-tocopherol concentrations have been associated with preterm delivery and low infant birthweight [6]. Lower infant α-tocopherol concentrations are associated with hemolytic anemia in low-birth-weight infants, lung inflammation with elevations in neutrophils and pro-inflammatory cytokines, and the development of neonatal lung disease such as bronchopulmonary dysplasia (BPD) [7,8,9]. Furthermore, fetal storage of α-tocopherol tends to occur in the third trimester of pregnancy. This may explain why preterm infants are born with lower body concentrations than term infants, all while facing a larger postnatal oxidative stress burden [8].

Research has generally reached a consensus on the antioxidant activity of α-tocopherol, but our understanding of γ-tocopherol is much more varied. Several studies suggest that α- and γ-tocopherol have opposing anti-inflammatory properties, with γ-tocopherol exerting mostly pro-inflammatory activity in human tissues [10]. Indeed, γ-tocopherol has been shown to increase in vivo inflammation by promoting lymphocyte migration [10], and higher γ-tocopherol concentrations have been associated with inflammatory conditions like osteoarthritis and asthma [11,12,13]. The pro-inflammatory actions of γ-tocopherol appear to relate to increased IFN-γ activity and increased cytokine production (IL-2, IL-4) [1]. Meanwhile, α-tocopherol exerts anti-inflammatory effects by modulating the signaling pathways involved in the expression of pro-inflammatory genes [2]. The opposing antioxidant properties of α- and γ-tocopherol further relate to their unique biochemical structures [14]. All tocopherol isoforms contain a chromane ring with a hydroxyl group and a hydrophobic side chain for penetration into biological membranes. α-tocopherol has additional methyl groups near its chromanol ring that allow for resonance stabilization and free radical reduction [14].

In general, tocopherols are important micronutrients found in a variety of dietary sources. Nuts, seeds, vegetable oils, and green leafy vegetables are all good sources of vitamin E, especially the α-tocopherol isoform. The current recommended dietary allowance (RDA) of vitamin E is 15 milligrams (mg)/day, measured exclusively as its α-tocopherol isoform. There are no current recommendations for appropriate amounts of other isoforms. As a whole, the American diet is heavily influenced by processed oils rich in γ-tocopherol (i.e., wheat germ, soybean, canola, corn, and vegetable oils) [15]. Individuals, specifically pregnant women, also tend to consume additional nutrients in daily multivitamins, which typically contain only the α-tocopherol isoform at a dose of around 90% of their RDA [16].

In maternal–fetal environments, nutrient absorption and subsequent biological activity are also regulated and affected by the placenta. The placenta is a vital organ that provides nutrients, oxygen, and waste product removal for the developing fetus via the umbilical vessels. Some nutrients are transferred from mother to baby via facilitated diffusion. For larger, less membrane-permeable substances, ionic gradients regulate transporter proteins and allow nutrients to cross from the placenta to the baby’s umbilical vessels [17]. Vitamin E is one such compound that transfers via a membrane protein, referred to as α-tocopherol transfer protein (α-TTP) [18,19,20]. Initial research into the transfer of tocopherol isoforms in maternal–fetal environments demonstrates lower umbilical cord concentrations of vitamin E isoforms compared to maternal plasma concentrations [21]. However, to our knowledge, placental concentrations of vitamin E isoforms have not yet been described. Through investigation of this unique organ, there is an opportunity to gain insights into the dynamics of maternal–fetal nutrition status, which may later allow for nutritional interventions that enhance body concentrations and perinatal health.

The purpose of this study is to evaluate the concentration of vitamin E isoforms, including α-tocopherol and γ-tocopherol, in placental tissue and its relationship with maternal and umbilical cord plasma concentrations. Seeking to understand more about dietary intake, nutrition status, and overall health in vulnerable populations, this study explores tocopherol concentrations and the process of nutrient transfer in mother–infant dyads.

## 2. Materials and Methods

Following Institutional Review Board (IRB) approval through the University of Nebraska Medical Center (IRB #112-15-EP), 82 mother–infant pairs were recruited at the time of delivery from the Labor and Delivery Unit, Newborn Nursery, and Level III academic Neonatal Intensive Care Unit (NICU). Infants made wards of the state or those with a prenatal diagnosis of congenital abnormalities or maternal gastrointestinal, liver, or kidney disease that affects nutrient metabolism were excluded. At delivery, maternal and cord blood plasma specimens were collected, as well as approximately 1 cm^3^ cross-sections of placental tissue. Specimens were protected from heat and light and promptly frozen at −80 °C within 12 h of clinical procurement, pending bulk analysis.

### 2.1. Quantification of Plasma and Placental Tocopherol Concentrations

All samples were analyzed at the Harvard School of Public Health Nutritional Biomarker Lab. Measurements of α-tocopherol and γ-tocopherol were obtained in cord blood samples, maternal plasma samples, and maternal placenta samples. Individual placenta sample weights were recorded in grams (g). Then, samples were homogenized in distilled, deionized water using mechanical pulverization (Polytron PT1200, Kinematica AG, Lucerne, Switzerland) to create an aqueous slurry. Both placenta and plasma samples were mixed in a 1:1 ratio of 250 μL plasma to 250 mL ethanol with 10 μg rac-tocopherol/mL as an internal standard. Both placenta and plasma samples were then extracted with 4 mL hexane, evaporated to dryness under nitrogen, and reconstituted in 100 mL ethanol-dioxane and 150 mL acetonitrile. Biological concentrations of tocopherols in the samples were quantified by high-performance liquid chromatography (HPLC) on a Restek Ultra C18 150 mm, 4.6 mm column with a 3 μm particle size encased in a column oven (Hitachi L-2350, Hitachi, San Jose, CA, USA). This equipment prevents temperature fluctuations and is equipped with a trident guard cartridge system (Restek, Corp., Bellefonte, PA, USA). A mobile phase was analyzed with a mixture of acetonitrile, tetrahydrofuran, methanol, and a 1% ammonium acetate solution (68:22:7:3) at a flow rate of 1.1 mL/min with a Hitachi L-2310 pump in isocratic mode, a Hitachi L-2455 diode array detector (300 nm and 445 nm), and a Hitachi L-2200 auto-sampler with water chilled tray. Four control samples were analyzed with each run for internal quality control. External quality control was monitored by participation in the standardization program for tocopherol analysis from the National Institute of Standards and Technology USA. Concentrations of α- and γ-tocopherol were reported in micrograms/gram (µg/g) for placental tissue samples and in micrograms/liter (µg/L) for maternal and umbilical cord plasma samples.

### 2.2. Evaluation of Dietary Nutrient Intake

At the time of delivery, mothers completed the Harvard Food Frequency Questionnaire (FFQ), a comprehensive dietary intake survey that has been validated in all adults and pregnant women [22]. It has greater utility over other methods of nutrient intake assessment (i.e., 24-h recalls) as it is more reflective of intake over time. Longitudinal dietary intake is calculated using questionnaire responses and the established nutrient content of selected foods, a process completed by trained personnel at the Harvard School of Public Health. Vitamin E intake is reported as milligrams/day of individual tocopherol isoforms. Maternal intake adequacy of α-tocopherol was assessed using the Institute of Medicine’s daily recommendation of ≥15 mg/day [5].

### 2.3. Collection of Other Demographic Data

Demographic information was collected on the maternal–infant pair using the electronic medical record. Maternal age and pre-pregnancy body mass index (BMI, in kilograms/meters^2^ (kg/m^2^)) were recorded. Characteristics from the time of delivery included mode of delivery (cesarean section vs. vaginal birth), infant gestational age, and birth weight in grams (g). Birth weight metrics were plotted on the 2013 Fenton growth chart for infants <37 weeks gestation or the World Health Organization 0–2 year growth chart for infants if ≥37 weeks gestation, and subsequent percentile rankings were recorded [23].

### 2.4. Statistical Analysis

Descriptive statistics were generated for each variable of interest, including medians and interquartile ranges (IQR) for continuous variables and frequencies and percentages for categorical variables. Non-parametric statistical testing, including the Mann–Whitney U test and Kruskal–Wallis test, compared continuous data between groups. Spearman’s correlation coefficients were utilized to relate maternal plasma, cord plasma, and placental tissue tocopherol concentrations. All dataset analysis was completed using SPSS version 29.0 (IBM Corporation, Armonk, NY, USA). A *p*-value < 0.05 was considered statistically significant.

## 3. Results

The median maternal age was 29.0 years, with a median maternal pre-pregnancy BMI of 28.9 kg/m^2^. A total of 80.5% of infants were born via vaginal delivery. The median birth gestational age was 39.7 weeks, and 2.4% of infants were born preterm (CGA < 37 weeks). A total of 8.5% of infants required NICU admission, and maternal complications like gestational diabetes and pre-eclampsia were found in 7.3% and 1.2% of mothers, respectively. The median total maternal dietary tocopherol intake was 24.7 mg/day, and 52.4% of mothers met the RDA for adequate vitamin E intake. For mothers meeting the RDA, the median α-tocopherol intake was 22.9 mg/day, compared to 10.7 mg/day for mothers who did not meet the RDA. Additional demographic data are included below in Table 1.

The median placental concentrations for α- and γ-tocopherol (µg/g) were 1.44 and 0.46, respectively. Median maternal tocopherol plasma concentrations (µg/L) were higher than umbilical cord tocopherol concentrations (µg/L) for both α-tocopherol and γ-tocopherol (*p*-values < 0.001, Table 2). The ratio of median α-tocopherol concentrations in maternal plasma compared to umbilical cord blood was 6.25, meaning maternal plasma contained approximately 6.25 times the amount of α-tocopherol compared to umbilical cord blood. Maternal plasma contained even higher concentrations of γ-tocopherol compared to umbilical cord blood, with a maternal–cord ratio of 9.08 (Table 2).

In all biological samples, α-tocopherol comprised a higher tocopherol proportion compared to γ-tocopherol. The median α:γ tocopherol ratio was significantly higher in umbilical cord blood compared to placenta (13.2 vs. 2.9; *p* < 0.001). α:γ tocopherol ratios between maternal plasma and placenta were also significantly different (9.9 vs. 2.9; *p* < 0.001). Maternal plasma and umbilical cord blood did not differ significantly in α:γ tocopherol ratios (*p*-value = 0.08) despite umbilical cord blood containing a higher ratio of α:γ tocopherol at 13.2 (Table 3).

The concentration of α-tocopherol in maternal plasma demonstrated a significant positive correlation with total maternal tocopherol intake (R = 0.26, *p* = 0.02). γ-tocopherol intake was significantly associated with α-tocopherol concentrations in maternal plasma (R = 0.24, *p* = 0.03). There were no significant correlations between total or isoform-specific intake and γ-tocopherol concentrations in the biological tissues. However, γ-tocopherol concentrations demonstrated significant positive correlations between all three biological sample types. The strongest relationship was observed between γ-tocopherol concentrations in maternal plasma and placenta tissue (R = 0.61, *p* <0.001). There were no significant correlations for concentrations of α-tocopherol within maternal plasma, umbilical cord blood, or placenta (Table 4).

## 4. Discussion

This study shows significant correlations between γ-tocopherol concentrations among maternal–fetal samples and demonstrates a significantly lower α- to γ-tocopherol ratio in placental tissue compared to maternal and umbilical cord plasma. Our previous studies have concluded the higher maternal–fetal transfer of α-tocopherol compared to γ-tocopherol and have described the subsequent impacts on clinical outcomes [3,4]. This study expands our knowledge of maternal–fetal transfer dynamics by making comparisons between proportions of α- and γ-tocopherol within placental tissue, as well as maternal and umbilical cord plasma. To our knowledge, our study is the first to report correlations among the major tocopherol isoforms within maternal blood, umbilical cord blood, and placenta. In this way, this study further characterizes vitamin E transfer dynamics in maternal–fetal environments, paving the way for additional studies into these mechanisms and clinical impacts.

### 4.1. Dietary Intake

Only 52.4% (n = 43) of mothers met the RDA for adequate intake of vitamin E, with a median α-tocopherol intake of 15.7 mg/day. The subset of mothers who failed to meet the RDA for vitamin E intake had a low median α-tocopherol intake of only 10.7 mg/day. This relatively high proportion of dietary inadequacy is consistent with prior studies in both American and Asian women [24,25]. This study also demonstrated significant positive, though weak, correlations between maternal plasma concentrations of α-tocopherol and maternal total tocopherol and γ-tocopherol intake. This contrasts with several other studies that failed to detect significant associations between tocopherol intake and serum tocopherol concentrations in various European cohorts [26,27,28,29]. However, these studies were not specific to pregnant women and often adjusted for additional factors like age, smoking status, alcohol consumption, and serum lipid concentrations. Our study did not demonstrate significant relationships between dietary intake and tocopherol isoforms in umbilical cord blood or placenta tissue. Nearly half of the mothers in our study had inadequate tocopherol intake, and it is possible that reduced tocopherol intake affects the balance between maternal utilization of this nutrient and its transfer to the growing fetus. Furthermore, pregnancy is an inflammatory state [30], and it is plausible that the biological utilization of vitamin E as a potent antioxidant may differ during pregnancy and affect serum and tissue concentrations. Our cohort of pregnant women also had a median pre-pregnancy BMI in the overweight range (BMI 28.9 kg/m^2^). Studies have shown individuals with greater adiposity tend to have lower circulating concentrations of vitamin E isoforms, possibly due to increased consumption from weight-related oxidative stress [31,32]. Overall, these findings promote further research into dietary and inflammatory confounders that may affect vitamin E metabolism, such as smoking status and obesity, and suggest a possible mechanism for tocopherol transfer in maternal–fetal environments that accounts for more than isoform intake.

Our study also demonstrated a proportional intake of γ-tocopherol, accounting for around 34% of total maternal tocopherol intake, with a median γ-tocopherol intake of 8.5 mg/day and a median total tocopherol intake of 24.7 mg/day. Overall, there is no established recommendation for γ-tocopherol intake nor any previous research into standardized intake proportions in comparison to α-tocopherol. However, there is evidence that γ-tocopherol is an increasingly prevalent isoform in the average American diet, given its presence in plant oils within more processed foods [33]. Specifically, the concentration of γ-tocopherol is highest in corn and soybean oils, and concentrations exceed the α-tocopherol content due to increased loss of the α-tocopherol isoform during oil refining processes [34,35]. In our study, we found greater α-tocopherol intake compared to γ-tocopherol intake in our cohort of pregnant women. This may be explained by additional dietary supplementation of the α-tocopherol isoform via prenatal vitamins in this cohort, a factor we did not separately analyze. Additionally, our study failed to demonstrate significant correlations between dietary tocopherol intake and the subsequent concentration of γ-tocopherol in multiple biologic tissues. These findings may be explained by the competitive binding and absorption of α- and γ-tocopherol in hepatic tissue. Both isoforms competitively bind to the same tocopherol transfer protein. Typically, α-tocopherol is preferentially bound, leaving more γ-tocopherol free floating in the plasma [36]. This process creates an inverse association between α-tocopherol intake and biologically available γ-tocopherol. It is also possible that there is a differential metabolism of tocopherol isoforms during the pro-inflammatory state of pregnancy that affects tissue storage and concentrations [30].

### 4.2. Plasma Concentrations

Concentrations of γ-tocopherol in maternal plasma, umbilical cord blood, and placenta demonstrated moderately strong and significant positive correlations with one another. In contrast, there were no significant correlations for α-tocopherol concentrations among the biological samples. These findings are consistent with previous reports depicting the correlations of α- and γ-tocopherol concentrations in maternal plasma and umbilical cord blood. For example, Yeum et al. reported a high correlation between cord and maternal γ-tocopherol concentrations with no significant correlation for α-tocopherol [37]. Kiely et al. studied a larger sample size and concluded a significant relationship between γ-tocopherol in maternal and cord plasma [38]. As aforementioned, these increased γ-tocopherol concentrations in plasma may be partially explained by the stronger competitive binding of α-tocopherol with plasma transfer proteins, which creates higher free-floating γ-tocopherol concentrations [36]. Furthermore, γ-tocopherol is known to have less affinity for tissue receptors like α-TTP, which may prevent it from being absorbed and utilized by bodily tissues [39]. Knowing this raises significant questions about the clinical impact of higher plasma γ-tocopherol, as this unbound form may be biologically unavailable.

Explanations for the lack of significance of α-tocopherol correlations across biological samples are twofold. First, research into tocopherol metabolites like carboxyethyl hydroxychromans (CEHCs) demonstrated elevated concentrations of these compounds in cord blood, which implies fetal or maternal metabolism of vitamin E [40]. This may explain the lack of significant correlations for parent compounds in maternal–fetal blood samples. Second, as alluded to previously, the relative affinity of α-tocopherol for both plasma transport and tissue receptor proteins may allow greater absorption and metabolism compared to the γ-tocopherol isoform. Despite these hypotheses, the exact explanation for the variability in correlations of tocopherol isoforms in biological samples is unclear. Further research is warranted into tocopherol receptors, metabolites, and in vivo activity.

### 4.3. Placenta Concentrations

There were lower α- to γ-tocopherol ratios in the placenta compared to maternal or umbilical cord blood, which may suggest increased accumulation of the γ-tocopherol isoform within placental tissue. Furthermore, the higher maternal–cord ratio for γ-tocopherol compared to α-tocopherol suggests less direct transfer of this isoform from maternal to fetal circulation and would support this same theory. Overall, there is limited literature on γ-tocopherol concentrations in human tissues, and the focus has mainly been on adipose and epithelial tissues [41]. To our knowledge, this is the first study to evaluate tocopherol isoforms in placental tissue and demonstrate lower ratios of α- to γ-tocopherol in this biological sample. Despite the lack of previous investigation into γ-tocopherol in the placenta, existing literature has documented descriptive evidence of the transport of α-tocopherol in the placenta, which occurs via serum lipoproteins and α-TTP [18,19,20]. Overall, studies have further demonstrated an inefficient transfer rate of α-tocopherol in the placenta, hypothesized to be due to low expression of lipoprotein or α-TTP in placental tissue [18,42,43,44]. Interestingly, γ-tocopherol is believed to have a lower affinity for α-TTP [39], which calls into question the apparent higher absorption observed in our study. Therefore, it is unlikely that γ-tocopherol outcompetes α-tocopherol for placental absorption via α-TTP. It is possible that there are additional placenta receptors other than α-TTP that preferentially allow for γ-tocopherol absorption. Additionally, the placental metabolism of α-tocopherol could explain the lower concentration of this isoform in placental tissue. Although research to date has been unable to quantify metabolites like CEHCs or the enzymes involved in tocopherol metabolism within placental tissue [40,45], the research overall is too limited to draw conclusions.

Another possible explanation for the increased proportion of γ-tocopherol in placental tissue may relate to the preferential transfer of α-tocopherol to fetal tissues. Although we did not directly analyze fetal tissue in this study, the reduced maternal–cord ratio for α-tocopherol compared to γ-tocopherol supports this hypothesis. α-tocopherol is a known antioxidant, and the fetal environment is often uniquely and negatively affected by oxidative stress. In fact, previous studies suggest that lower infant α-tocopherol concentrations are associated with worsening oxidative stress and poorer outcomes, including intraventricular hemorrhage, bronchopulmonary dysplasia, neurodevelopmental delay, and stunting of growth [31,46]. It is possible that the maternal–fetal environment values the antioxidant properties of α-tocopherol and preferentially passes this isoform to the developing fetus in utero. Furthermore, postnatal studies on maternal breast milk support preferential transfer of α-tocopherol compared to γ-tocopherol and promote significantly greater proportions of the α-tocopherol isoform in early breast milk, also known as colostrum, which may serve to mediate postnatal inflammation in the newborn [47].

By quantifying tocopherol isoforms in placental tissue, our findings raise further questions regarding their metabolism and biological implications within the maternal–fetal environment. Placental dysregulation and oxidative stress have consistently been suspected of causing many of the common problems in pregnancy. Indeed, oxidative stress within placental tissue has shown frequent correlation and possible causation with multiple adverse outcomes like pre-eclampsia, premature delivery, intrauterine growth restriction, low birth weight, spontaneous abortion, and neonatal demise [48,49,50,51,52]. As outlined previously, there is well-established evidence for the role of vitamin E isoforms in mediating inflammation and oxidative stress. In relation to the maternal–fetal environment, studies have demonstrated improved placental function and lower oxidative stress burden, as measured by various biochemical markers, in high-risk mothers who were supplemented with vitamin E during pregnancy [53]. However, this study only supplemented with the α-tocopherol isoform of vitamin E and failed to measure isoform concentrations in placental tissue or assess the role of other isoforms like γ-tocopherol on placental inflammation. This omission of γ-tocopherol analysis is critical, as there is mixed evidence regarding the exact pro- or anti-inflammatory nature of this tocopherol in maternal–fetal environments. Prior studies have associated increased maternal serum γ-tocopherol concentrations with preterm birth and chorioamnionitis, while other studies have shown positive correlations with γ-tocopherol and better Apgar scores and neonatal growth parameters [6,7,54]. Additionally, Thoene et al. analyzed relationships between tocopherol isoforms and inflammatory compounds in mother–infant dyads and discovered a significant negative association between the concentration of γ-tocopherol in placental tissue and the concentration of pro-inflammatory IL-8 in maternal serum [3]. Taken in context with our findings demonstrating increased γ-tocopherol concentrations in placental tissue, further exploration of the clinical outcomes related to placental tocopherol concentrations is warranted. We suggest that future research in this area is needed to enhance our understanding of the placental metabolism and transport of γ-tocopherol, along with its clinical implications.

### 4.4. Strengths and Limitations

The strengths of the current study include its novel assessment of tocopherol isoforms in placental tissue, an area of perinatal nutrition that has not yet been studied. Our evaluation of a population (n = 82) with a complete collection of paired placental tissue, maternal and umbilical cord blood, and maternal dietary information allowed for stronger analysis and enhanced understanding of vitamin E transfer dynamics in maternal–infant dyads.

Our study has multiple limitations. We did not evaluate tocopherol isoform concentrations alongside total lipid or inflammatory concentrations in individual patients. Due to the transport of plasma lipoproteins, total tocopherol concentrations are known to be affected by abnormal lipid concentrations [20,55]. We also did not adjust for other demographic variables that may influence metabolism, nutrient concentration, or dietary intake, such as obesity [31,32], smoking status [56,57], or medication use [58,59]. We acknowledge that this may have impacted our results. Additionally, most (97.6%) of the above data are from term-delivered mother–infant dyads, which makes it challenging to generalize our findings to the preterm population most impacted by low antioxidant capacity. Subsequent studies should aim at including preterm infants, as known variation exists in transfer rates of nutrients among these populations. Further, our maternal–infant population had low rates of perinatal complications (pre-eclampsia, NICU admission, etc.), which prevented analysis of placental tocopherol concentrations between distinct clinical outcome groups. Our study population also included a large proportion of mothers with inadequate α-tocopherol intake; however, it is important to note that this may reflect existing perinatal populations, as high dietary inadequacy of vitamin E has been observed in multiple other studies [24,25].

## 5. Conclusions

This study demonstrates a correlation between maternal dietary intake of both total tocopherols and γ-tocopherol and maternal plasma concentrations of α-tocopherol only. γ-tocopherol was the only isoform to exhibit significant correlations among all biological samples. Most notably, the ratio of α:γ-tocopherol demonstrates tissue-specific variation and is lowest in placental tissue compared to maternal or umbilical cord blood. More research is needed to identify the relationship of these findings to clinical outcomes in the mother and offspring, and this research would benefit from studying a larger maternal–infant population with higher rates of perinatal complications.

## Figures and Tables

**Table 1:** Maternal and Infant Demographics.

Variable	Median	Interquartile Range
Maternal age (years)	29.0	24.0–33.0
Maternal pre-pregnancy BMI (kg/m^2^)	28.9	22.9–32.6
Birth gestational age	39.7	39.0–40.6
Infant birth weight (g)	3523	3295.5–3830.5
Maternal total tocopherol intake (mg/day)	24.7	18.6–32.3
Maternal α-tocopherol intake (mg/day)	15.7	10.9–23.6
Maternal γ-tocopherol intake (mg/day)	8.5	6.6–12.4

**Table 2:** Median Tocopherol Concentrations and Interquartile Range within Placenta, Maternal, and Umbilical Cord Plasma.

	Placenta (µg/g)	Maternal Plasma (µg/L)	Umbilical Cord Plasma (µg/L)	Ratio of Maternal–Cord Tocopherol Concentrations
α-tocopherol	1.44 (0.41–2.37)	17,892.9 (14,910.2–20,699.7)	2861.8 (2061.1–3600.4)	6.25
γ-tocopherol	0.46 (0.30–0.70)	1783.7 (1310.8–2469.1)	196.4 (147.6–295.9)	9.08

**Table 3:** α:γ-Tocopherol Ratios within Placenta, Maternal, and Umbilical Cord Plasma.

	Placenta	Maternal Plasma	Umbilical Cord Plasma
α:γ-tocopherol	2.9	9.9	13.2
			*p*-value (Kruskal–Wallis Test)
Placenta with Maternal Plasma			<0.001
Placenta with Umbilical Cord Plasma			<0.001
Maternal Plasma with Umbilical Cord Plasma			0.08

**Table 4:** Spearman’s Correlation Coefficients of Tocopherol Concentrations for Maternal Dietary Intake and Placenta, Maternal, and Umbilical Cord Plasma Concentrations.

		α-Tocopherol	γ-Tocopherol
Total tocopherol intake with:	Maternal Plasma	0.26 *	0.06
	Placenta	0.08	0.02
	Umbilical Cord	0.13	−0.06
α-tocopherol intake with:	Maternal Plasma	0.20	−0.10
	Placenta	0.11	−0.14
	Umbilical Cord	0.16	−0.15
γ-tocopherol intake with:	Maternal Plasma	0.24 *	0.10
	Placenta	0.06	0.04
	Umbilical Cord	0.04	−0.03
Maternal Plasma with Umbilical Cord Plasma		−0.15	0.46 **
Maternal Plasma with Placenta		0.09	0.61 **
Placenta with Umbilical Cord Plasma		0.20	0.47 **

* Indicates *p*-value < 0.05; ** Indicates *p*-value < 0.001.

## Data Availability

The data presented in this study are available on request from the corresponding author. The data are not publicly available due to ethical considerations regarding participant confidentiality.
